# Low dose aspirin blocks breast cancer-induced cognitive impairment in mice

**DOI:** 10.1371/journal.pone.0208593

**Published:** 2018-12-10

**Authors:** Adam K. Walker, Aeson Chang, Alexandra I. Ziegler, Haryana M. Dhillon, Janette L. Vardy, Erica K. Sloan

**Affiliations:** 1 Drug Discovery Biology Theme, Monash Institute of Pharmaceutical Sciences, Monash University, Parkville, Victoria, Australia; 2 Neuroscience Research Australia, Randwick, New South Wales, Australia; 3 School of Psychiatry, University of New South Wales, Randwick, New South Wales, Australia; 4 Division of Cancer Surgery, Peter MacCallum Cancer Centre, East Melbourne, Victoria, Australia; 5 Centre for Medical Psychology & Evidence-based Decision-Making, School of Psychology, Faculty of Science, University of Sydney, Camperdown, New South Wales, Australia; 6 Concord Clinical School, Sydney Medical School, University of Sydney, Camperdown, New South Wales, Australia; 7 Concord Cancer Centre, Concord Repatriation General Hospital, Concord, New South Wales, Australia; 8 Cousins Center for PNI, UCLA Semel Institute, Jonsson Comprehensive Cancer Center, and UCLA AIDS Institute, University of California Los Angeles, Los Angeles, California, United states of America; University of Alabama at Birmingham, UNITED STATES

## Abstract

Cancer patients with non-central nervous system tumors often suffer from cognitive impairment. While chemotherapy has long been attributed as the cause of these memory, learning and concentration difficulties, we recently observed cognitive impairment in cancer patients prior to treatment. This suggests the cancer alone may be sufficient to induce cognitive impairment, however the mechanisms are unknown. Here, we show that we can experimentally replicate the clinical phenomenon of cancer-associated cognitive impairment and we identify inflammation as a causal mechanism. We demonstrate that a peripheral tumor is sufficient to induce memory loss. Using an othotopic mouse model of breast cancer, we found that mice with 4T1.2 or EO771 mammary tumors had significantly poorer memory than mice without tumors. Memory impairment was independent of cancer-induced sickness behavior, which was only observed during the later stage of cancer progression in mice with high metastatic burden. Tumor-secreted factors were sufficient to induce memory impairment and pro-inflammatory cytokines were elevated in the plasma of tumor-bearing mice. Oral treatment with low-dose aspirin completely blocked tumor-induced memory impairment without affecting tumor-induced sickness or tumor growth, demonstrating a causal role for inflammation in cognitive impairment. These findings suggest that anti-inflammatories may be a safe and readily translatable strategy that could be used to prevent cancer-associated cognitive impairment in patients.

## Introduction

Cognitive impairment is widespread in cancer patients with peripheral solid tumors, with 70% of patients reporting cognitive symptoms and 40% showing measureable impairment in learning, concentration, executive function and memory [1–3]. Impairment in cognition in cancer survivors has been reported up to 20 years after cancer treatment. Coupled with the long survival rate for individuals with common types of curable cancer (e.g., breast and colorectal cancer), sustained impairment means that we face a pressing public health demand to understand the mechanisms of cancer-associated cognitive impairment and to develop treatment options that improve the quality of life for cancer survivors.

While cancer-related cognitive impairment has long been attributed to the effect of chemotherapy—colloquially termed ‘chemobrain’—compelling evidence has emerged to indicate that cancer patients present with cognitive impairment *prior* to cancer treatment. We identified cognitive impairment in colorectal cancer patients prior to chemotherapy, neoadjuvant chemoradiation or surgery [3]. Cognitive changes have also been described in breast cancer patients prior to treatment [4–6]. Cognitive impairment may contribute to misconceptions that patients can hold about their illness, which may lead to inaccurate perceptions of the intent of treatment and poor medical decision-making during consultation with physicians [7–10]. The observation of cognitive impairment in cancer patients prior to treatment suggests a need for early intervention for cognitive impairment. To address this, it is important to understand how non-central nervous system (CNS) solid tumors such as colorectal and breast cancer induce cognitive impairment.

While it has been suggested that the stress or anxiety of a cancer diagnosis may contribute to cognitive impairment, learning- and memory-related behavioral changes have been identified in tumor-bearing mice and rats that do not experience diagnosis anxiety [11–13]. This suggests that factors other than psychological distress contribute to cancer-associated cognitive impairment prior to treatment. A possible candidate mechanism for cancer-associated cognitive impairment is neuroinflammation. Inflammation is a hallmark of cancer [14] and the link between inflammation and changes in learning and memory have been well characterised in non-cancer settings [15–17]. A number of studies provide correlative support for this hypothesis by showing that increased circulating inflammatory markers coincide with changes in cognition and fatigue in breast cancer patients [4, 18–20]. However, in a number of these studies cognition was assessed after surgical resection of the tumor [18, 20], which elevates inflammation and could plausibly result in cancer-associated cognitive impairment. Animal models have been used to interrogate the contribution of specific aspects of the cancer journey to cancer-associated cognitive impairment. As such, links have been identified between inflammation and cancer-associated cognitive impairment in rodents with cancer but that have not undergone surgery [12, 13, 21–25]. However, a causal role of inflammation in cancer-associated cognitive impairment has not been demonstrated. Targeting inflammation with a non-steroidal anti-inflammatory drug has been shown to alleviate symptoms of depression and fatigue [26], but the effect on cognition is unknown. Furthermore, no study has investigated cognitive impairment in a metastatic cancer model, which means that it is unknown whether the magnitude of cancer burden predicts the magnitude of cognitive impairment.

Here we test the hypothesis that a peripheral solid tumor can induce cognitive impairment as demonstrated by memory deficits using two syngenic, orthotopic mouse models of breast cancer. We test the hypothesis that inflammation plays a role in cancer-associated cognitive impairment by examining if the anti-inflammatory non-selective cyclo-oxygenase (COX) inhibitor aspirin is sufficient to abrogate tumor-induced cognitive impairment.

## Materials and methods

### Animals and ethics

BALB/c and C57BL/6J female mice (8–12 weeks old) (Monash University, Australia) were housed individually in standard shoebox cages in a temperature and humidity controlled environment with a 12/12-h modified dark-light cycle (lights on at 19:00). Food and water were available *ad libitum*. Mice were randomly allocated to experimental groups. Blood was collected by post-mortem cardiac puncture and tissues were removed after perfusion with sterile PBS. All procedures involving mice were carried out under protocols approved by the Monash University Animal Ethics Committee (protocol number MIPS2015.01) and in accordance with National Health and Medical Research Council guidelines. Animals were monitored daily to ensure they were not experiencing distress. Humane endpoints based on body weight loss, body condition scoring and metastatic progression were in place. Mice were euthanized with CO_2_. No unexplained mortality occurred in these studies.

### Breast cancer models

To investigate cognitive function in response to an orthotopic primary tumor, 1 x 10^5^ 4T1.2 tumor cells [27] syngenic to BALB/c mice or EO771.lmb tumor cells [28] syngenic to C57BL/6J mice were injected in 20 μL PBS (Invitrogen, USA) into the left 4^th^ mammary fat pad of anesthetized (3% isoflurane) mice. Control non-tumor bearing mice received injections of 20 μL PBS. Cell line identity was confirmed by short tandem repeat profiling (Cellbank, Australia). Control mice received 20 μL PBS injected into the left 4^th^ mammary fat pad under anaesthesia. Primary tumor growth was monitored by digital caliper twice a week and volume calculated using the formula: (length x width^2^)/2. Distant metastasis was monitored twice a week by bioluminescence imaging under anaesthesia. Tumor cells were transduced to express codon-optimized luciferase and either mCherry or eGFP under control of the ubiquitin C promoter [29, 30]. Mice were injected with *d*-luciferin (150 mg/kg, CHOICE Analytical) via tail vein and primary tumor burden and metastasis was quantified using an IVIS Lumina II (Perkin Elmer) [31, 32]. Cells were determined to be mycoplasma free before injection using a MycoAlert Mycoplasma Detection Kit (Lonza, Australia) or using primers as previously described [33]. Non-tumor bearing mice that received PBS underwent identical procedures as tumor bearing mice including anaesthesia and imaging.

### Tumor cell-conditioned medium experiments

To explore how peripheral tumors signal to the brain, we investigated if factors secreted by tumor cells are sufficient to induce cognitive impairment. Tumor cell-conditioned medium was prepared as previously described [34]. 4T1.2 cells at 80–90% confluency were incubated in DMEM with low glucose and free of serum, pyruvate, L-glutamine, HEPES and phenol red (Sigma-Aldrich). After 12 h, tumor cell-conditioned medium was collected and filtered (0.42 μm filter). Control medium was prepared using the same protocol but in the absence of tumor cells. BALB/c mice were injected (IP) daily with 400 μl control or tumor cell-conditioned medium, as described.

### Anti-inflammatory treatment

BALB/c mice were treated with either asprin in drinking water (Aspro, Bayer Australia Ltd) (0.125 μg/ml) or water (control) beginning 36 h prior to tumor cell injection and continuing throughout the experiment. Water was changed every 12 h and liquid consumption quantified. Mice were pre-determined to drink an average of 4 ml/day resulting in an estimated dose of 25 mg/kg/day. This dose has been shown to produce a similar pharmacodynamic effect on COX-1 inhibition as a dose of 100 mg/day in humans, which is considered a low dose [35].

### Behavioral assays

All behavioral experiments were performed during the dark phase when mice are most active. Experimenters were blinded to treatment conditions during behavioral testing. Mice were handled and trained on the burrowing task for 1 week prior to experimentation. Sickness (body weight loss, burrowing activity) were measured as described previously [36]. Burrowing is a measure of activity with a motivational component and was examined by placing PVC pipes (20cm length) containing 200g of food pellets into the home cage of each mouse. Mice were allowed 30 minutes to burrow and remove pellets from the pipe which is an instinctive behavior. After 30 minutes the pellets remaining in the tube were weighed and the percentage of pellets removed was calculated.

Memory was assessed using the novel object/novel place recognition test. Novel object/novel place recognition was conducted under low lighting. To acclimate to the arena, mice were exposed to the arena 2–3 times for 5 minutes and then underwent habituation to the novel object/novel place recognition task 1–2 times prior to tumor cell injection. Briefly, mice were placed into a 40 cm x 40 cm arena that had spatial cues on the walls and contained two identical objects. Mice were allowed to explore for 5 minutes, then returned to their home cage for 5 minutes and the objects cleaned with ethanol and dried. One of the identical now-familiar objects was exchanged with a novel object that was placed in a different location within the arena. Mice were then returned to the arena to explore freely for an additional 5 minutes. Viewer III (Biobserve GmbH, Bonn, Germany) software was used to objectively analyse distance travelled and time spent exploring the novel object. Virtual squares were drawn closely around each object as a region of interest. The software was programmed to recognise when the mouse moved inside a region of interest corresponding to either the old or the new object. Time spent within each region of interest was recorded. Novel object recognition was calculated by determining the percentage of time spent with the novel object divided by the total time exploring all the objects in the arena. Mice were excluded if they spent less than 20 seconds exploring the familiar objects in the learning phase. Mice were tested within the first week of tumor growth (4T1.2: day 4 or 5, E0771 day 7) and again in the second week (4T1.2: day 11, E0771: day 15), and after 8 days of tumor cell-conditioned medium injection.

Locomotor activity was assessed for 5 minutes in complete darkness under infrared lighting in a 40 cm x 40 cm arena. Distance travelled was assessed using Viewer III (Biobserve GmbH, Bonn, Germany) software.

### Cytokine analysis

A panel of 23 cytokines was measured, using a Bioplex Pro mouse cytokine 23-plex kit (Bio-Rad, Gladesville, Australia), according to the manufacturer’s protocol. Plasma samples of 4T1.2 tumor bearing and non-tumor bearing mice 30 days after tumor cell injection were analysed undiluted. The plate was read using a Bio-Rad BioPlex200 machine. Median fluorescence data are collected by the instrument and the Bio-Plex Manager software calculates cytokine concentrations as pg/ml based on the standard curve per cytokine using a 5-parameter logistic method.

### Statistical analysis

One-way or two-way repeated measures analyses of variance (ANOVA) (tumor vs. no tumor) were used to analyse longitudinal data from the novel object/novel place recognition test, burrowing test, body weight measurements, primary tumor growth and metastatic progression where appropriate. The effect of soluble factors and intervention with aspirin on cognitive impairment was assessed for single time-point data (locomotor activity, spleen weight, novel object/novel place recognition, cytokines) using two-way between-subject ANOVA or t-test. Spearman’s correlations were used to investigate the relationship between cancer burden at day 26 and sickness behavior measured by burrowing at day 28. Planned comparisons were performed to assess change from the control group, and were determined using Newman-Keuls multiple comparisons tests or t-tests with the alpha level of 0.05 adjusted for the number of comparisons to control for family-wise error. Experiments were conducted in duplicate or triplicate.

## Results

### Mammary adenocarcinoma causes memory impairment

**Fig 1:**
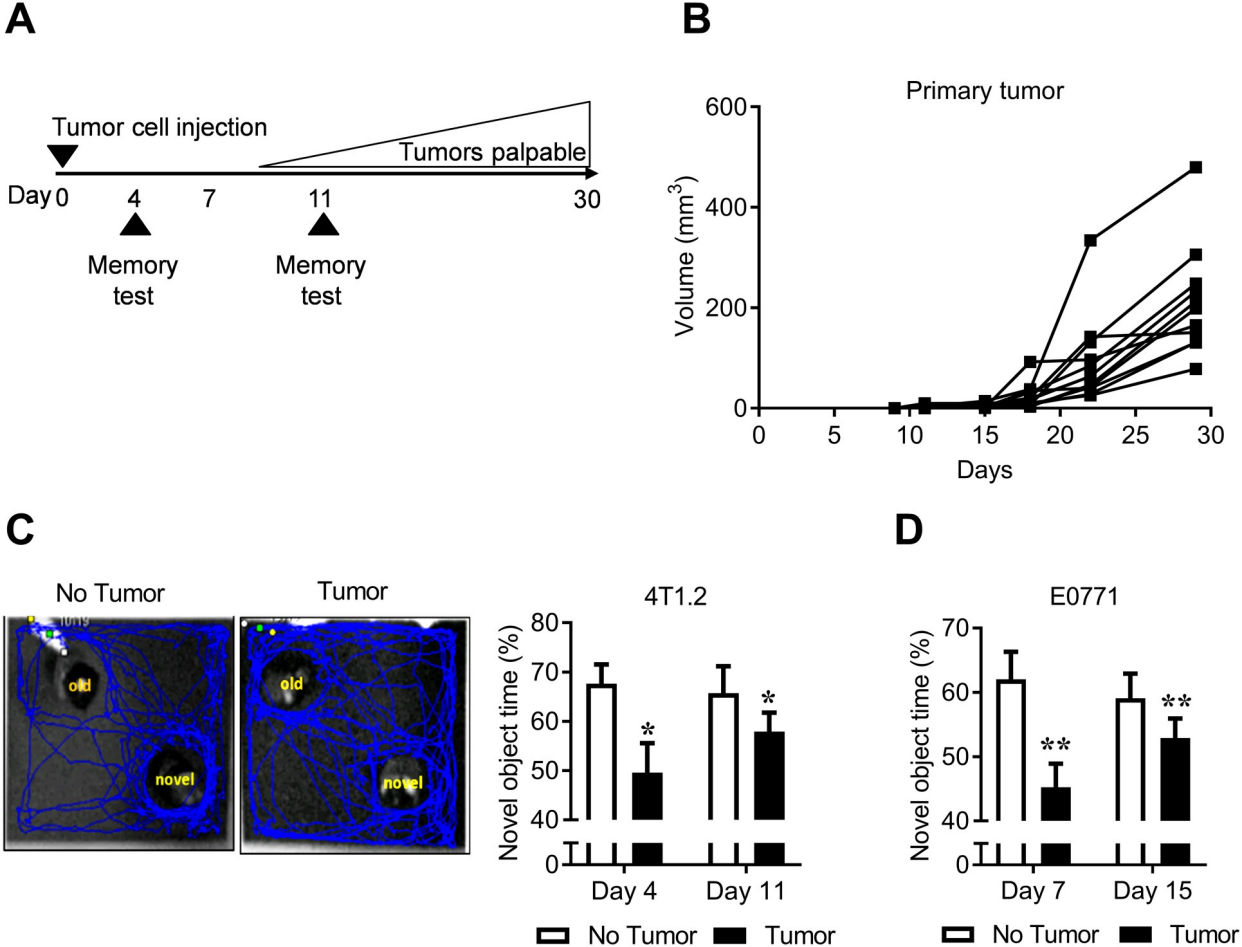
Mammary tumors induce memory impairment. ***A*** Experimental design to evaluate the effect of a primary mammary tumor on cognitive impairment. ***B*** 4T1.2 primary tumor growth measured by caliper. Individual mice are shown. ***C*** Memory was assessed in 4T1.2 tumor-bearing (n = 26) and non-tumor (n = 23) mice in the test phase of the novel object/novel place recognition test. Representative images and quantified data are shown (mean ± SE). The blue lines represent where the mouse has travelled over the course of 5 minutes. ***D*** Quantified data (mean ± SE) of EO771 tumor (n = 9) and non-tumor (n = 10) bearing mice in the test phase of the novel object/novel place recognition test 7 and 15 days after tumor cell injection.*p < 0.05; **p < 0.01.

To explore the effect of cancer on cognitive function, we used an orthotopic mouse model of breast cancer to investigate if the presence of a mammary tumor was sufficient to induce memory impairment (Fig 1A). Mice were injected with 4T1.2 tumor cells into the mammary fat pad and primary tumors became palpable after 9 days (Fig 1A and 1B). To assess the effect of a primary tumor on memory, we used the novel object/novel place recognition test. Novel object/novel place recognition measures memory by capitalizing on the innate preference of mice to explore novelty, and requires mice to remember *what* they saw and *where* they saw it in a previous period of exploration. To achieve this, mice explore two identical objects, experience a rest period, and then have a choice of exploring either one of the previously explored objects or a novel object. Memory is assessed by the time spent exploring the novel object compared to the previously explored object.

Mice with tumors showed impaired memory within four days of tumor growth, several days before the tumor became palpable (*F*_(1,47)_ = 5.75, *p* < 0.05) (Fig 1C). Memory impairment was sustained for more than 11 days after tumor cell inoculation (Fig 1C). Analysis of memory over the first two weeks of tumor growth found the magnitude of memory impairment was not associated with the increase in tumor burden. To determine if tumor-induced memory impairment occurs independently of cell line or mouse strain, we investigated the effect of EO771 mammary tumors on memory in C57BL/6J mice. EO771 tumor cells were injected into the mammary fatpad of C57BL/6J mice and memory function was assessed using the novel object/novel place recognition test on days 7 and 15 after tumor cell injection. Consistent with the finding that the 4T1.2 cell line in Balb/c mice impairs memory (Fig 1C), EO771 tumors in C57BL/6J also impaired memory performance within 7 days of tumor growth and this was sustained for more than 15 days (*F*_(1,17)_ = 8.46, *p* < 0.001) (Fig 1D).

### Cancer-induced cognitive impairment is independent of sickness behavior

**Fig 2:**
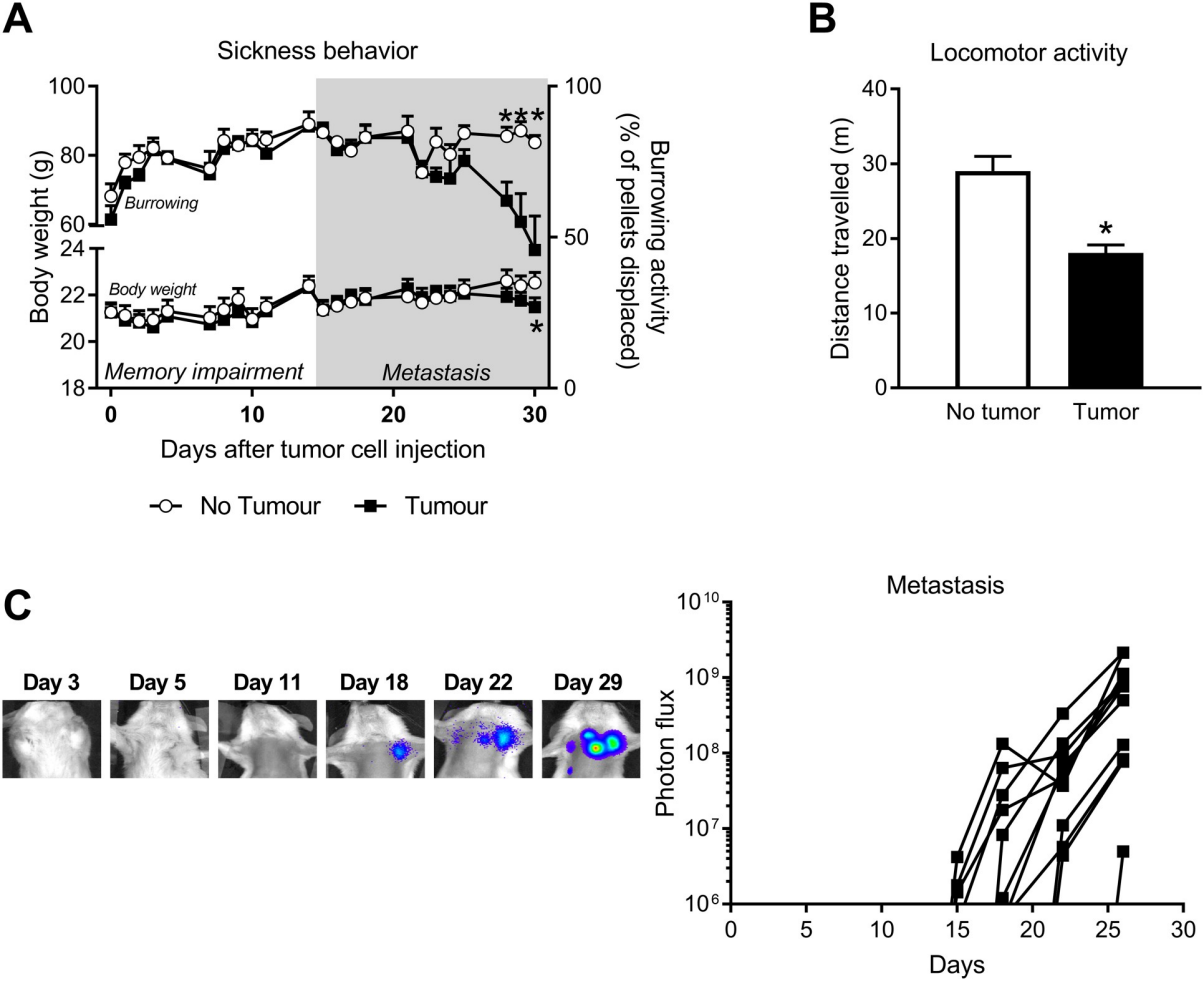
Memory impairment during the early stage of tumor growth is independent of sickness behavior. ***A*** Body weight (mean ± SE) and burrowing activity (mean ± SE) for non-tumor-bearing mice (n = 12) and 4T1.2 tumor-bearing mice (n = 12) from 0 to 30 days after tumor cell injection. ***B*** Locomotor activity as measured by distance travelled (mean ± SE) for non-tumor (n = 8) and 4T1.2 tumor-bearing (n = 15) mice 24 days after tumor cell injection. ***C*** Representative images and quantified data of metastasis tracked using bioluminescence imaging for individual mice (n = 12). *p < 0.05.

Chronic diseases including cancer have been linked to sickness behavior, which manifests as reduced food intake (weight loss), reduced activity and social engagement, and increased fatigue [37]. As the novel object/novel place recognition test relies on engagement with objects, interpretation of the findings may be confounded by factors that reduce activity and therefore exploration and object engagement. However, at the time that cognitive deficits were detected (day 4 and 11 of tumor growth) there was no evidence of reduced activity (Fig 2A); tumor-bearing mice and control mice spent the same total amount of time exploring the identical objects that were originally placed in the arena, regardless of tumor model (*p* > 0.05).

Assessment of weight loss and burrowing in mice with 4T1.2 tumors found evidence of sickness behavior late in the disease trajectory (Fig 2A), three weeks after detection of cognitive impairment (Fig 1C). Body weight loss began after 28 days of tumor growth and reached significance at 30 days (*p* < 0.05)(Fig 2A). Burrowing activity began to significantly decline after 23 days of tumor growth (*F*_(1,399)_ = 4.35, *p* = 0.04) and locomotor activity measured after 24 days was significantly lower for tumor-bearing mice (*t*_(21)_ = 2.13, *p* = 0.045) (Fig 2B). To explore if sickness behavior was associated with metastasis, we used bioluminescence imaging to non-invasively determine the kinetics of tumor cell dissemination (Fig 2C). We found that sickness behavior occurred only during the metastatic phase of disease that was first detectable between days 15 and 18 of tumor growth (Fig 2C), and was associated with increased tumor burden (*r* = -0.65, *p* = 0.02). These findings suggest that cancer-induced sickness behavior is temporally distinct from cancer-induced cognitive impairment and explains why attempts to assess memory on day 18 (data not shown) were unsuccessful due to the inactivity of the mice.

To rule out the possibility that cognitive impairment was due to immune activation against the fluorescent or bioluminescent proteins that were used to tag cells to detect tumor progression [38], we investigated if untagged 4T1.2 tumor cells affect cognition. Untagged tumors cells were injected into the fourth mammary fatpad and the novel object/place test was used to examine memory. Mice with untagged tumors had impaired cognition compared to non-tumor bearing control mice (S1A Fig). Cognition was impaired to similar levels as in mice with tagged tumors (*p* > 0.05), suggesting that the tumor itself is responsible for cognitive impairment, rather than an effect of the experimental system used to monitor tumor progression.

### Soluble factors secreted from tumor cells induce cognitive impairment

**Fig 3:**
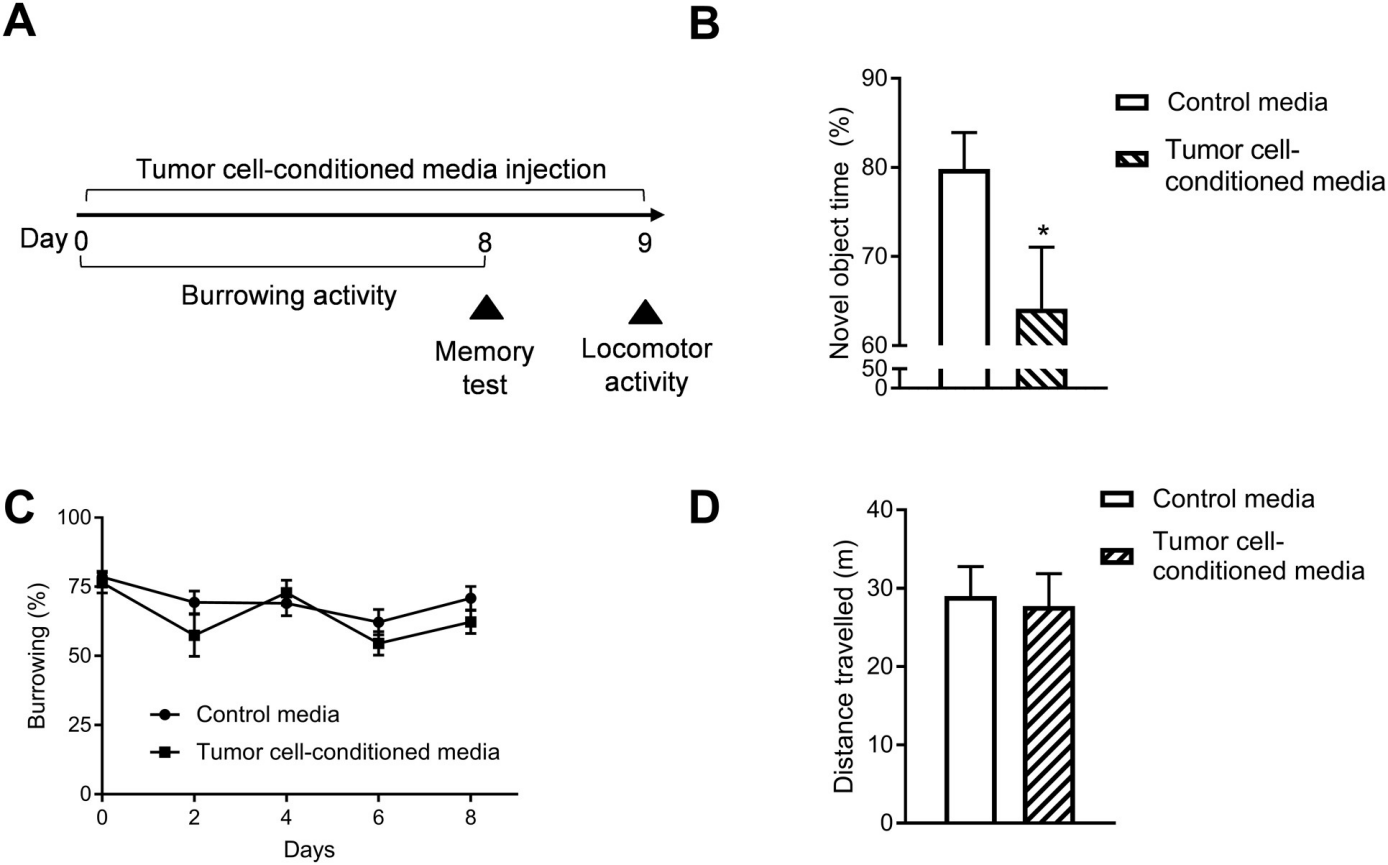
Soluble factors secreted from tumor cells are sufficient to induce cognitive impairment. ***A*** Experimental design to evaluate the effect of tumor-secreted soluble factors on cognitive impairment. ***B*** Time spent exploring the novel object (mean ± SE) for mice treated with control medium (n = 18) versus mice treated with 4T1.2 tumor cell-conditioned medium (n = 14). ***C*** Burrowing activity (mean ± SE) for mice treated with control medium (n = 19) versus mice treated with tumor cell-conditioned medium (n = 14). ***D*** Locomotor activity as measured by distance travelled (mean ± SE) for mice treated with control medium (n = 12) versus mice treated with tumor cell-conditioned medium (n = 14). *p < 0.05.

To explore how peripheral tumors signal to the brain to affect cognition, we investigated if factors secreted by tumor cells are sufficient to induce cognitive impairment. Mice were treated daily with medium conditioned by tumor cells (vs. non-conditioned medium) and memory was assessed using the novel object/novel place recognition test (Fig 3A). Tumor cell-conditioned medium contains factors that are secreted by the tumor cells. Treatment with tumor cell-conditioned medium resulted in memory deficits that were not observed in mice treated with non-conditioned medium (*t*_(29)_ = 2.22, *p* = 0.03)(Fig 3B). Treatment with tumor cell-conditioned medium did not affect body weight (*p* > 0.05), burrowing (Fig 3C) or locomotor activity (Fig 3D) throughout treatment (all *p* > 0.05), indicating that changes in memory were not secondary to cancer-related sickness. These findings suggest that soluble factors released by tumor cells are sufficient to induce cognitive impairment.

### Aspirin blocks tumor-induced cognitive impairment

To investigate if inflammatory factors that are released by tumor cells contribute to memory impairment, we first investigated if there was evidence of inflammation in mice with tumors.

**Fig 4:**
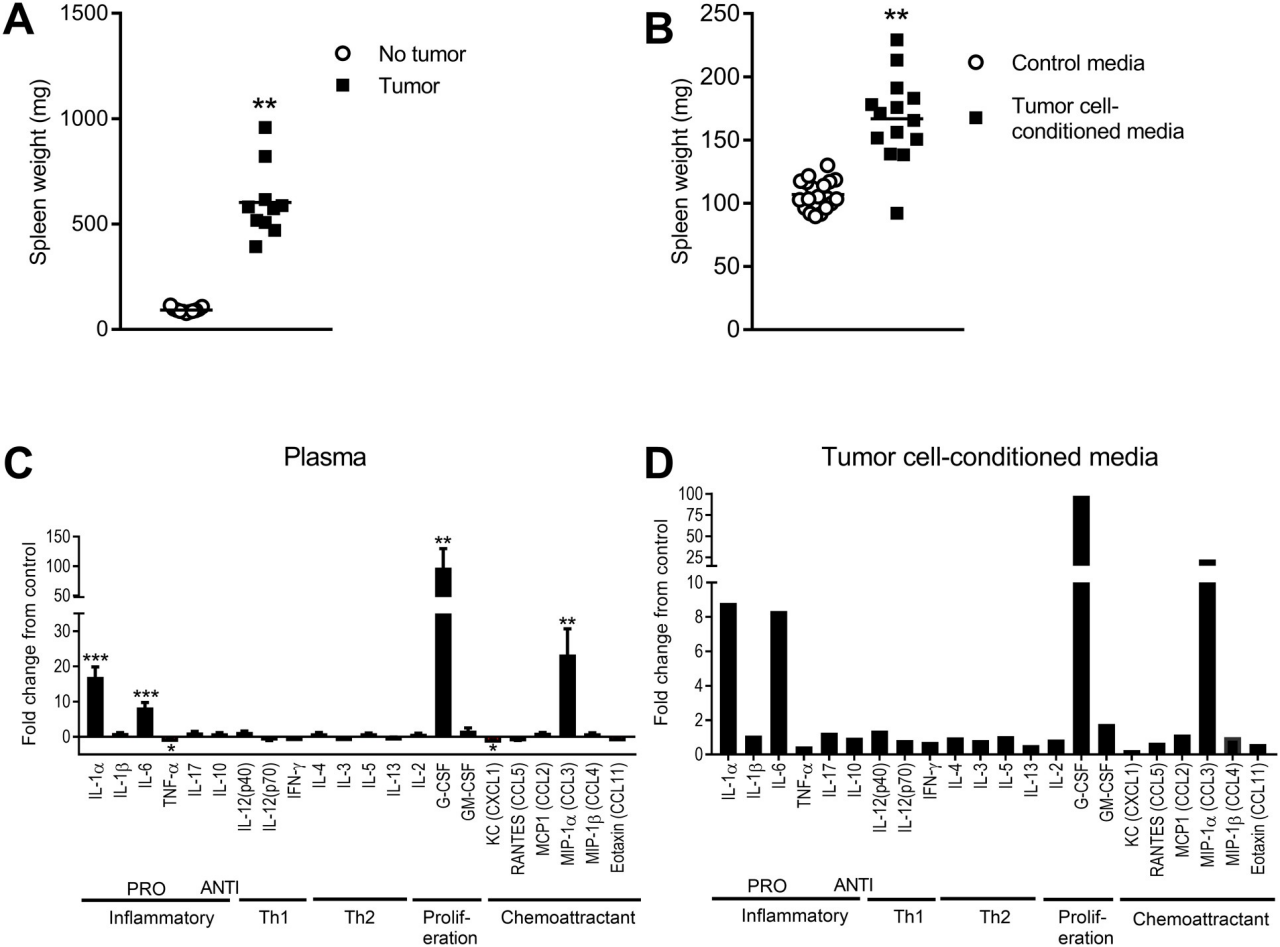
Tumor-bearing mice show evidence of inflammation. A Spleen weights (mean ± SE) of non-tumor mice (n = 12) and tumor-bearing mice (n = 10) after 24 days of tumor growth. B Spleen weights (mean ± SE) for mice treated with control medium (n = 19) versus mice treated with 4T1.2 tumor cell-conditioned medium (n = 14). C Cytokine and chemokine profile of plasma of tumor-bearing mice (n = 10–11) (mean ± SE). Fold change relative to non-tumor mice. D Cytokine and chemokine profile of 4T1.2 tumor cell-conditioned medium. Fold change relative to control medium. **p < 0.01.

We observed that tumor-bearing mice had larger spleens after 24 days of tumor growth (Fig 4A), compared with mice without tumors (*t*_(20)_ = 6.73, *p* < 0.001). Treatment with tumor cell-conditioned medium also induced splenomegaly (*t*_(31)_ = 7.21, *p* < 0.001) (Fig 4B). This is consistent with previous observations of splenomegaly in tumor-bearing mice, and has been linked to expansion of myeloid cells [39]. To define the secreted inflammatory profile induced by tumors we analysed plasma cytokine levels. Mice with tumors had elevated levels of inflammatory cytokines including IL-1α, IL-6, MIP-1α and G-CSF and lower levels of TNFα and CXCL1 compared to non-tumor bearing mice (all *p* < 0.05; statistics are provided in S1 Table) (Fig 4C). To determine if tumor cells are a source of these cytokines, we analysed the effect of treatment with tumor cell-conditioned medium on cytokine levels and found a very similar inflammatory profile with elevated IL-1α, IL-6, MIP-1α and G-CSF (Fig 4D).

**Fig 5:**
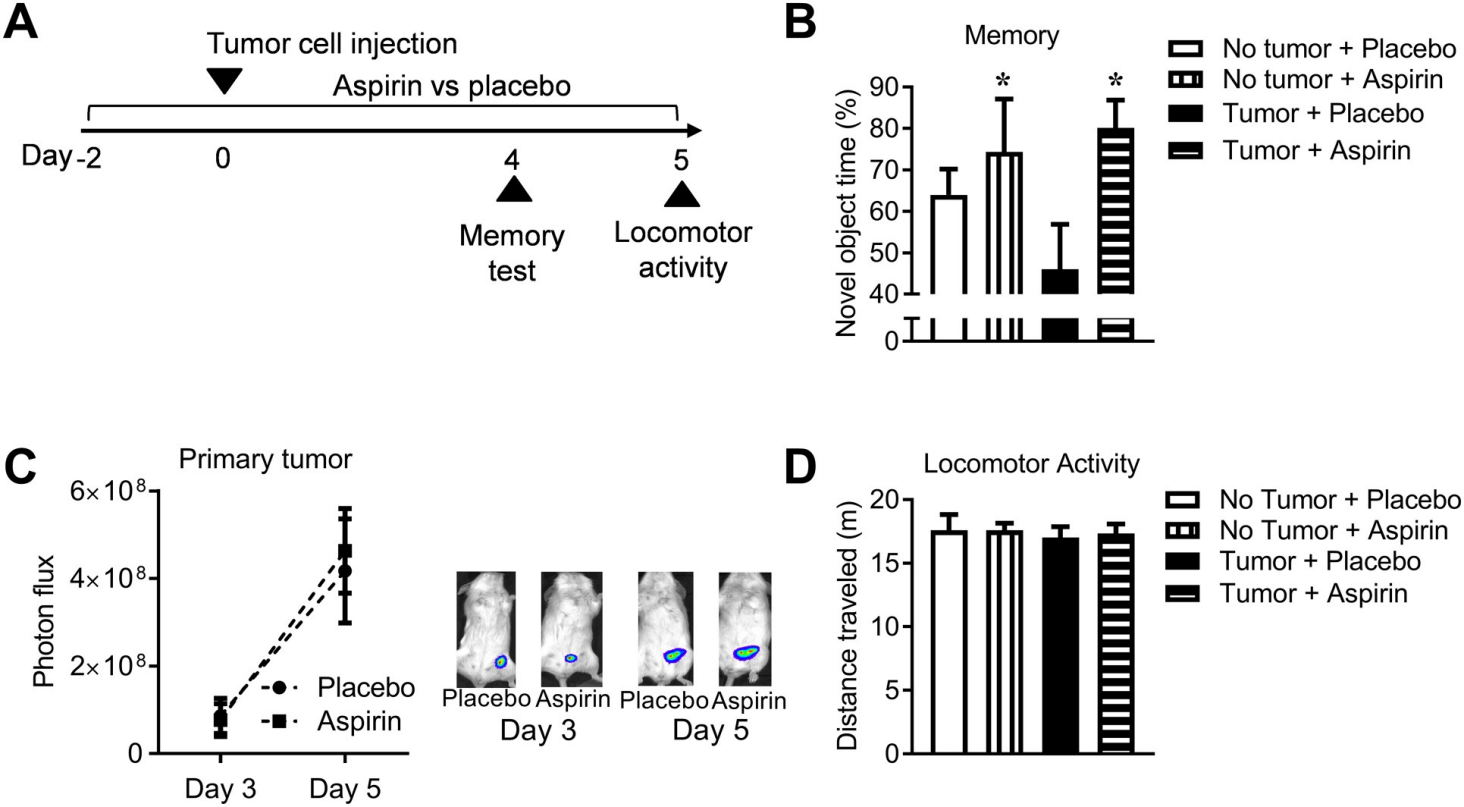
The anti-inflammatory drug aspirin blocks tumor-induced cognitive impairment. ***A*** Experimental design. ***B*** Quantified data (mean ± SE) of 4T1.2 tumor bearing mice treated with (n = 9) and without (n = 11) aspirin vs non-tumor bearing mice treated with (n = 8) and without (n = 11) aspirin in the test phase of the novel object/novel place recognition test 4 days after tumor cell injection. ***C*** Representative images and quantified data of primary tumor growth measured by bioluminescence imaging (mean ± SE) of tumor bearing mice treated with placebo (n = 14) vs aspirin (n = 10). ***D*** Locomotor activity as measured by distance travelled (mean ± SE) for tumor bearing mice treated with (n = 9) and without (n = 11) aspirin vs non-tumor bearing mice treated with (n = 8) and without (n = 11) aspirin. *p < 0.05.

To determine if inflammation has a causal role in cancer-induced cognitive impairment, we treated mice with the non-steroidal anti-inflammatory drug (NSAID) aspirin to block inflammation and examined the effect on cancer-induced memory impairment (Fig 5A). As studies have indicated that NSAIDs such as aspirin may have cancer preventative effects [40], we used low dose aspirin to reduce the potential for aspirin to impede tumor development. Aspirin improved memory function in control and tumor bearing mice as measured using the novel object/novel place recognition test, (*F*_(1,35)_ = 5.59, *p* = 0.02) (Fig 5B). Treatment with aspirin did not affect primary tumor growth or metastasis (*p* > 0.05), showing that the improvement in memory was not secondary to changes in tumor burden (Fig 5C). Locomotor activity was not affected by aspirin (*p* > 0.05), indicating no effect on sickness behavior (Fig 5D). These data indicate that aspirin may be able to prevent tumor-induced memory impairment, and may improve memory function in the absence of tumors.

## Discussion

This is the first study to show a causal role for inflammation in cancer-induced cognitive impairment. Here, we demonstrate that a solid peripheral tumor is sufficient to impair memory. This provides experimental evidence to support clinical observations that cognitive impairment in cancer patients may not be exclusively attributed to treatment, but may also be due to the cancer itself [3–6]. We demonstrate for the first time that soluble factors released by the tumor are sufficient to induce cognitive impairment and show that the NSAID aspirin prevents cancer-associated cognitive impairment. This provides a safe and readily translatable strategy that could be used to prevent cancer-associated cognitive impairment in patients.

Cognitive impairment was observed within a week of tumor cell inoculation, raising the possibility that patients with very early stage cancer may already exhibit symptoms of cognitive impairment. Studies have reported less than half of cancer patients accurately understand their prognosis and treatment intent, which impairs their medical decision-making rationale and may even lead to regret about treatment decisions [7, 9, 10]. Strategies to improve consultation practices have generally focused on development of physicians’ communication with patients [41]. These studies suggest pharmacological intervention may be an alternative strategy. We also found that tumor-induced cognitive impairment was sustained and did not increase with tumor burden suggesting that a low threshold of cancer burden is required to induce maximal cognitive impairment. Our recent clinical study also supports the finding that cancer burden is not related to the magnitude of cognitive impairment [3]. In a study of colorectal cancer patients, we found increased incidence of cognitive impairment compared to age-matched healthy controls, and no difference in the incidence of impairment between colorectal patients with and without non-CNS metastatic disease [3]. These findings suggest that cognitive impairment is likely to exist in many cancer patients at the time of diagnosis, and may be sustained in the absence of interventions that target the biological mechanisms of tumor-to-brain communication. Furthermore, these findings highlight the benefit of intervening early after cancer detection as, even during the very early stages of tumor growth, cognitive impairment was evident in tumor-bearing mice.

Identifying the cause of cognitive impairment in cancer patients has been challenging because memory deficits may be induced by treatment and by high levels of cancer burden as part of a cluster of sickness behaviors that include fatigue and inactivity [42]. This has made it difficult to distinguish the effects of cancer versus treatment, and explains why cancer-induced cognitive impairment was identified in cancer patients only very recently [3]. A strength of the current study is the capacity to distinguish sickness behavior from tumor-induced memory impairment by using a clinically relevant cancer model that replicated the entire disease trajectory from growth of a primary tumor in the orthotopic site to metastatic dissemination to distant organs. Additionally, use of non-invasive bioluminescence imaging provided a sensitive strategy to detect low levels of tumor cells at both the primary tumor site and during cancer spread; as few as 10^3^ cells which is 100-fold fewer than those injected to initiate the primary tumor (S1B Fig). This provided a unique opportunity to track cancer cell dissemination and quantify total cancer burden alongside the emergence of sickness behaviors. The finding that sickness behavior is associated with the magnitude of cancer burden but that cancer-induced memory impairment is independent of cancer burden, suggests that long-distance communication from the tumor to the brain may already be impairing memory function long before patients experience sickness-related symptoms.

In this study we used the novel object/novel place recognition task as a robust and validated measure of cognitive function in order to identify mechanisms of tumor-to-brain communication that impede cognitive performance. Performance in this task has been reported to rely heavily on the hippocampus and perirhinal cortex, two areas that are integral for memory and sensory processing [43, 44]. We chose this task because it taps into these integral regions of cognition, which allowed us to reliably observe cognitive changes in response to tumors, metastatic processes, tumor cell-conditioned medium and aspirin. Additional studies that use tests of cognitive function that rely on other brain regions will be important to determine if the effects of cancer are specific to those brain regions.

There is currently no definitive strategy for treating cognitive impairment in cancer patients. Current therapeutic approaches focus on psychological (e.g. adjustment/compensatory) and/or behavioral (e.g. cognitive rehabilitation) intervention [45]. However, these strategies are unlikely to block tumor-to-brain inflammatory signalling, which may explain our recent failure to improve objective cognitive function through web-based cognitive rehabilitation [46]. The findings presented here suggest that anti-inflammatory drugs such as aspirin may be a novel intervention strategy to treat cognitive impairment in cancer patients. Aspirin is an inexpensive, well-tolerated drug with the potential for rapid translation to target cancer-induced cognitive impairment in patients and supplement behavioral intervention strategies. Aspirin is safe in multiple cancer populations [47–49], and may have anti-cancer effects [50], in addition to reducing cognitive symptoms. For effective translation, it will be important to determine if cancer-induced cognitive impairment is reversible. As clinical studies have observed cognitive impairment in cancer patients at diagnosis [3, 51], treatment with anti-inflammatory drugs may need to be prophylactic if memory deficits cannot be reversed. It will be necessary to determine if aspirin protects against deficits in other cognitive domains, such as learning and concentration. Here we only examined memory using the novel object/novel place recognition assay because the aim was not to recapitulate the entire syndrome of cognitive symptoms seen in patients, but rather to use a robust, validated outcome measure of cognitive function in mice to interrogate mechanisms of cancer-associated cognitive impairment and identify a potential novel treatment. Future studies are needed to identify if other cognitive processes are involved.

Repurposing aspirin or other anti-inflammatory drugs to treat cancer-induced cognitive impairment would be a groundbreaking shift towards emphasising patient quality of life in cancer treatment. Randomised clinical trials are required to determine if aspirin is efficacious in clinical populations of patients without aspirin contraindications, and determine that aspirin does not interact negatively with current cancer treatments.

## Supporting information

S1 Fig ***A*** Quantified data (mean ± SE) of untagged 4T1.2 tumor (n = 17) and non-tumor (n = 23) bearing mice in the test phase of the novel object/novel place recognition test four days after tumor cell injection. ***B*** Representative images of wells seeded with 100–10,000 cells. Bioluminescence successfully detected 1000 cells. Quantified data of photon flux/sec indicate linear dose response (n = 2).
(TIF)

S1 Table Statistics of plasma cytokines in 4T1.2 tumor-bearing mice that significantly differed from non-tumor mice. (TIF)
