# Supplementary figures and images for: Low dose aspirin blocks breast cancer-induced cognitive impairment in mice
Source: PLoS One. 2018 Dec 10;13(12):e0208593. doi: 10.1371/journal.pone.0208593 (PMC6287899; doi:10.1371/journal.pone.0208593)

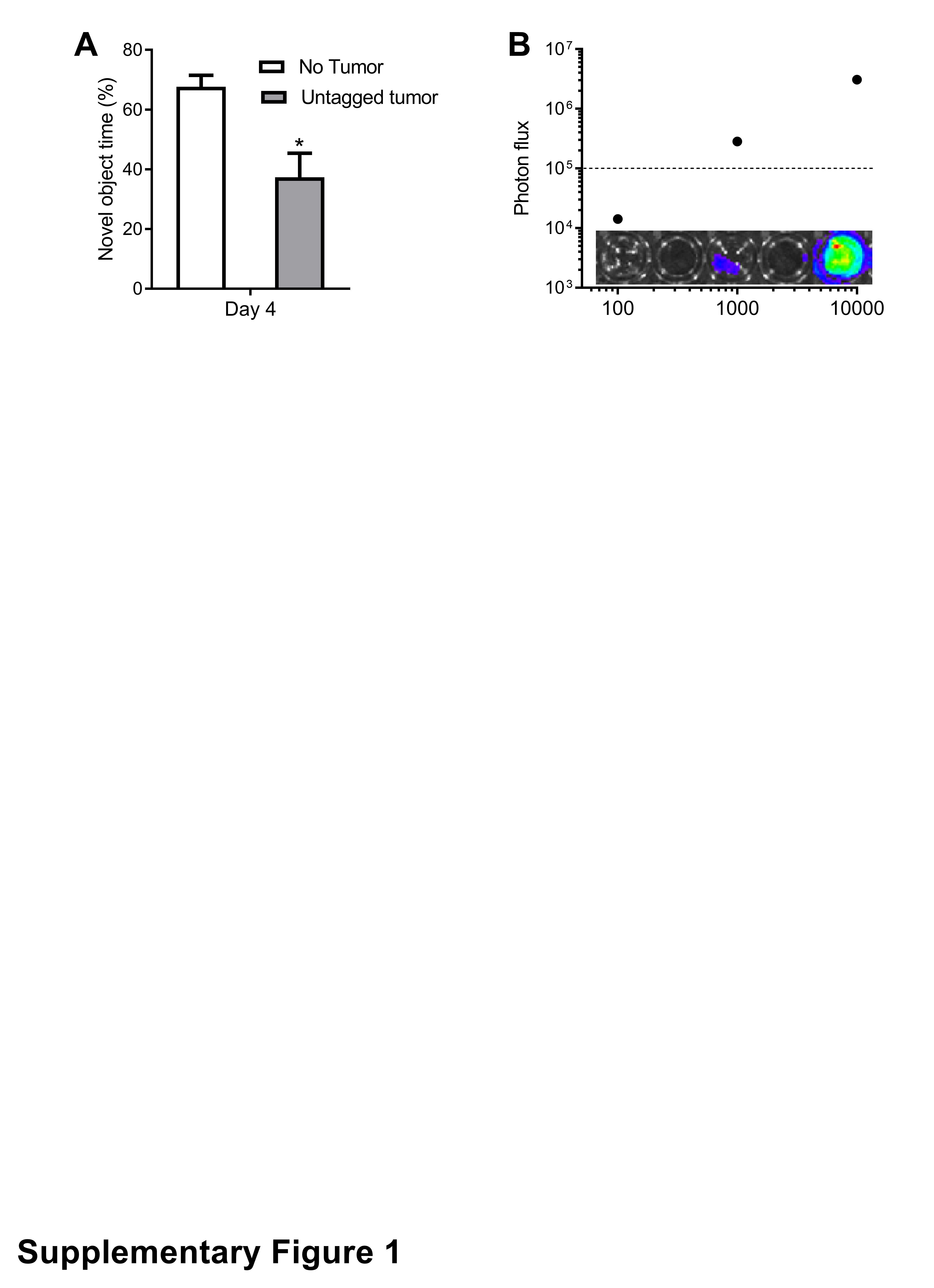

Supplement: S1 Fig — A Quantified data (mean ± SE) of untagged 4T1.2 tumor (n = 17) and non-tumor (n = 23) bearing mice in the test phase of the novel object/novel place recognition test four days after tumor cell injection. B Representative images of wells seeded with 100–10,000 cells. Bioluminescence successfully detected 1000 cells. Quantified data of photon flux/sec indicate linear dose response (n = 2). (TIF) [file pone.0208593.s001.tif]

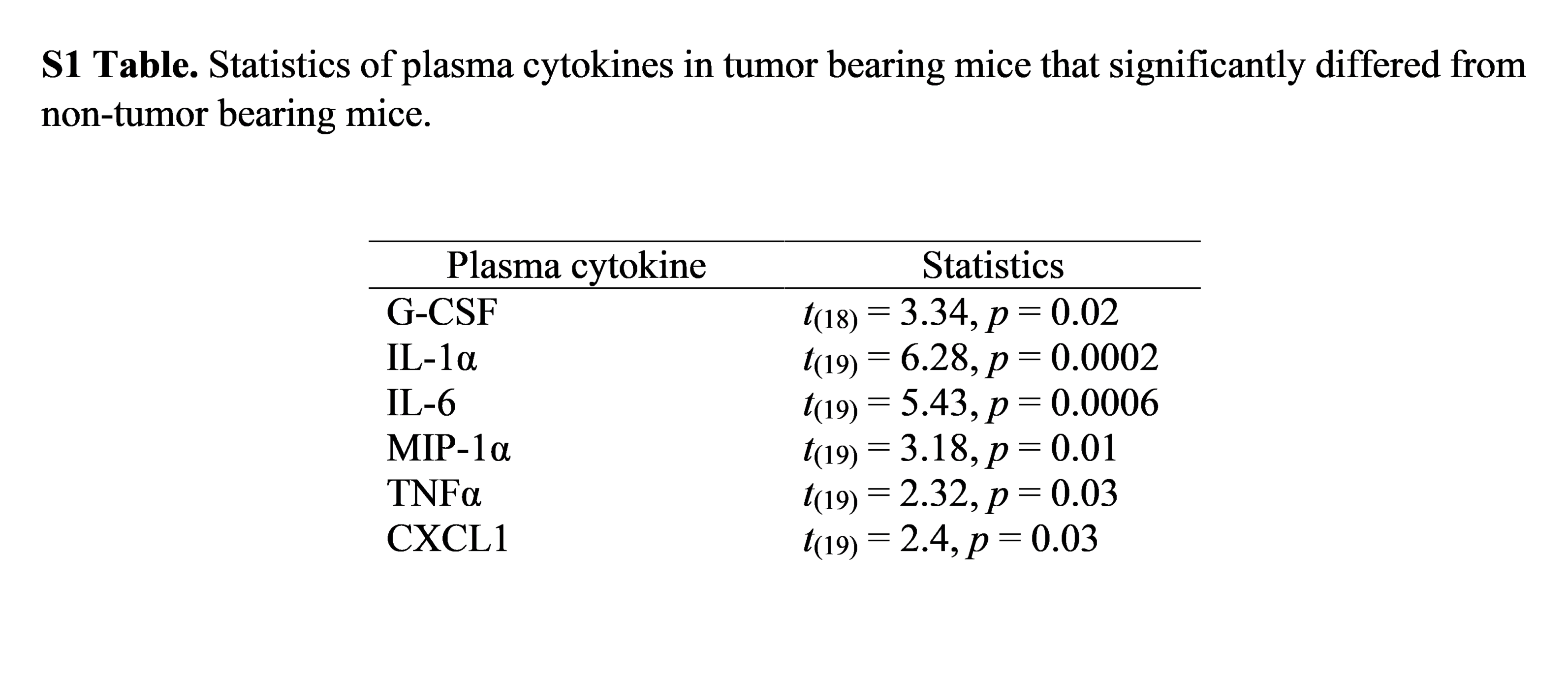

Supplement: S1 Table — (TIF) [file pone.0208593.s002.tif]
